# Genome-Scale Analysis Reveals Extensive Diversification of Voltage-Gated K^+^ Channels in Stem Cnidarians

**DOI:** 10.1093/gbe/evad009

**Published:** 2023-01-21

**Authors:** Adolfo Lara, Benjamin T Simonson, Joseph F Ryan, Timothy Jegla

**Affiliations:** Whitney Laboratory for Marine Bioscience, University of Florida, St Augustine, Florida, USA; Department of Biology and Huck Institutes for the Life Sciences, Penn State University, University Park, Pennsylvania, USA; Whitney Laboratory for Marine Bioscience, University of Florida, St Augustine, Florida, USA; Department of Biology, University of Florida, Gainesville, Florida, USA; Department of Biology and Huck Institutes for the Life Sciences, Penn State University, University Park, Pennsylvania, USA

**Keywords:** cnidarian, Kv, KCNQ, EAG, potassium channel, *Nematostella*

## Abstract

Ion channels are highly diverse in the cnidarian model organism *Nematostella vectensis* (Anthozoa), but little is known about the evolutionary origins of this channel diversity and its conservation across Cnidaria. Here, we examined the evolution of voltage-gated K^+^ channels in Cnidaria by comparing genomes and transcriptomes of diverse cnidarian species from Anthozoa and Medusozoa. We found an average of over 40 voltage-gated K^+^ channel genes per species, and a phylogenetic reconstruction of the Kv, KCNQ, and Ether-a-go-go (EAG) gene families identified 28 voltage-gated K^+^ channels present in the last common ancestor of Anthozoa and Medusozoa (23 Kv, 1 KCNQ, and 4 EAG). Thus, much of the diversification of these channels took place in the stem cnidarian lineage prior to the emergence of modern cnidarian classes. In contrast, the stem bilaterian lineage, from which humans evolved, contained no more than nine voltage-gated K^+^ channels. These results hint at a complexity to electrical signaling in all cnidarians that contrasts with the perceived anatomical simplicity of their neuromuscular systems. These data provide a foundation from which the function of these cnidarian channels can be investigated, which will undoubtedly provide important insights into cnidarian physiology.

**Significance**

The genome of the starlet sea anemone *Nematostella vectensis* revealed a surprising diversity of ion channels, raising the possibility that cnidarians might have complex electrical signaling despite having outwardly simple neuromuscular anatomy. Here, we show that all cnidarians have a high diversity of voltage-gated K^+^ channels, and we find evidence for 28 of these channels in stem cnidarians. This contrasts sharply with nine voltage-gated K^+^ channels predicted for stem bilaterians and suggests that unexpectedly diversified electrical signaling properties are a central requirement for cnidarian physiology.

## Introduction

**Fig. 1.**
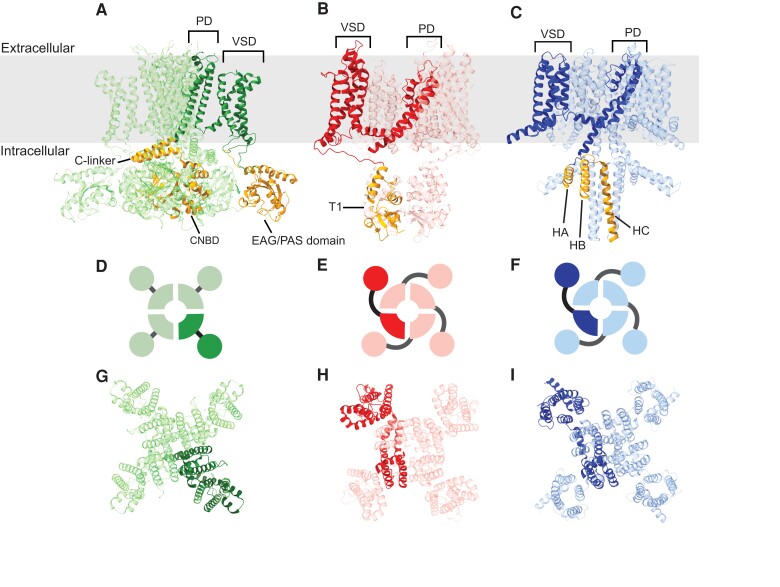
Animal voltage-gated K^+^ channels are comprised of three structurally distinct gene families. (*A*) Representative protein structures are shown for (*A*) EAG (mouse Eag1 [Whicher and MacKinnon 2016]), (*B*) Kv (rat Kv1.2 [Long et al. 2005]), and (*C*) KCNQ (frog KCNQ1 [Sun and MacKinnon 2017]). Structures are shown from a side view in the plane of the membrane (gray box) with a single subunit of the tetramer highlighted to illustrate the domain structure. Subunits from all three subfamilies share a transmembrane core consisting of a VSD and PD. Four independent VSDs surround the central PD. The channel families can be easily differentiated by their structurally distinct intracellular gating domains (gold highlights): the N-terminal EAG/PAS and C-terminal C-linker/CNBHD for EAG, the N-terminal T1 assembly domain for Kv, and the C-terminal HA-HC assembly domain for KCNQ. Extracellular views of the arrangement of subunits in the tetramers are shown for EAG (*D* and *G*), Kv (*E* and *H*), and KCNQ (*F* and *I*) in diagrammatic views (*D*–*F*) and structural views (*G*–*I*) with the same subunits highlighted as in *A*–*C*. Note that in Kv and KCNQ, the VSDs are domain-swapped relative to the PD, whereas in EAG, they are nonswapped.

Animal voltage-gated K^+^ channels comprise three highly conserved and molecularly distinct gene families, Shaker (Kv), KCNQ, and Ether-a-go-go (EAG). These channels fill a wide range of physiological roles, most notably regulating neuronal and muscular excitability by controlling action potential threshold and repolarization. All three voltage-gated K^+^ gene families encode structurally homologous tetrameric channels, with each subunit contributing one-fourth of a tetrameric pore domain (PD) and one of four independent voltage sensor domains (VSDs) that surround the PD (MacKinnon 1991; Long et al. 2005; Whicher and MacKinnon 2016; Sun and MacKinnon 2017). Individual subunits consist of six transmembrane domains (TMs), with the first four TMs (S1–S4) forming a complete VSD and the last two TMs (S5 and S6) contributing to the eight TM PD (fig. 1). Because of the deep evolutionary divergence of the Kv, KCNQ, and EAG gene families, sequence identity across this common structural core is much higher within gene families than across gene families, providing a robust ability to distinguish sequences belonging to each gene family via search algorithms such as BLAST (Wei et al. 1996; Jegla et al. 2009, 2012; Martinson et al. 2014; Li et al. 2015a, 2015b).

Furthermore, subunits encoded by each of the animal voltage-gated K^+^ channel families have differences in cytoplasmic domain content that serve as independent diagnostic identifiers. EAG gene family subunits can be definitively identified by the presence of a cytoplasmic domain homologous to cyclic nucleotide-binding domains (CNBDs; fig. 1*A*) attached to the pore via a helical domain known as the C-linker (Brams et al. 2014). These same features can be found in cyclic nucleotide-gated (CNG) channels (Kaupp et al. 1989), hyperpolarization-gated channels (HCN; Gauss et al. 1998; Ludwig et al. 1998; Santoro et al. 1998), plant voltage-gated K^+^ channels (Schachtman et al. 1992; Sentenac et al. 1992), ciliate protozoan K^+^ channels (Jegla and Salkoff 1995), and eubacterial cAMP-gated K^+^ channels (Brams et al. 2014), which together comprise the CNBD Superfamily of voltage-gated K^+^ channels (Brams et al. 2014; Jegla et al. 2018). Despite a common core domain arrangement, animal HCN and CNG channels are easily separated from EAGs because they share low amino acid identity with EAG channels and are not K^+^ selective. They, therefore, lack the consensus K^+^ selectivity sequence found in the PD of EAG channels (Whicher and MacKinnon 2016) and can also be distinguished by their high sequence identity to known HCN or CNG channels. It should be noted that EAG channels are not gated by cyclic nucleotides, and their CNBD is, therefore, sometimes referred to as a cyclic nucleotide-binding homology domain or CNBHD (Brelidze et al. 2012). This domain modulates EAG channel gating and appears to have an intrinsic protein ligand encoded within the channel sequence itself (Brelidze et al. 2012). The EAG family is divided into three functionally independent subfamilies (Eag, Erg, and Elk) (Warmke and Ganetzky 1994). Channel assembly is restricted to subunits within the same subfamily (Wimmers et al. 2001; Zou et al. 2003), although the molecular subunit sorting mechanism has not been characterized. Most EAG family channels also include a highly conserved N-terminal eag domain comprised in part by a Per-Arndt-Sim (PAS) fold (Cabral et al. 1998). However, the domain has been lost during evolution in some clades of Erg subfamily channels (Martinson et al. 2014), so it is not a reliable indicator for EAG family genes.

Kv channels in contrast can be definitively distinguished from EAG and KCNQ channels by the presence of their unique N-terminal cytoplasmic domain T1 (fig. 1*B*) which is evolutionarily related to BTB/POZ dimerization domains found in some transcription factors (Kreusch et al. 1998; Jahng et al. 2002). T1 forms a tetrameric ring hanging in the cytoplasm (Kobertz et al. 2000; Long et al. 2005) and mediates subfamily-specific assembly of channels (Shen and Pfaffinger 1995; Kreusch et al. 1998; Nanao et al. 2003). The Kv family comprises four gene subfamilies (Shaker, Kv1; Shab, Kv2; Shaw, Kv3; and Shal, Kv4) that encode functionally distinct channel types which can be expressed independently in the same cell (Covarrubias et al. 1991; Salkoff et al. 1992) based on this T1-restricted assembly (Shen and Pfaffinger 1995; Bixby et al. 1999). KCNQ channels in contrast, lack T1 and instead have a highly conserved and diagnostic coiled-coil domain in the C-terminus (fig. 1*C*) which appears to play a similar role as T1 in channel assembly (Schwake et al. 2006). KCNQ channels are unique among metazoan voltage-gated K^+^ channels in that they strictly require PIP2 to gate (Suh and Hille 2002; Zhang et al. 2003), but this function does not provide additional diagnostic sequence signatures.

Phylogenetic comparison of voltage-gated K^+^ channel genes from diverse bilaterians indicates that the last common bilaterian ancestor harbored just nine types of voltage-gated K^+^ channels. These include two KCNQ family channels (Li et al. 2015a), four Kv family channels, one each of Shaker, Shab, Shaw, and Shal (Jegla et al. 2012; Li et al. 2015a), and three EAG family channels (one each of Eag, Erg, and Elk; Martinson et al. 2014; Li et al. 2015b). The number of voltage-gated K^+^ channels in the genomes of bilaterian species range from as few as 9 in *Drosophila melanogaster* to 40 in human and mouse (Li et al. 2015a, 2015b), and even more in most bony fish (Jegla et al. 2009). There has been relatively little elaboration of the ancestral voltage-gated K^+^ channel set in most of the bilaterian genomes examined to date except for vertebrates, in which a series of whole-genome duplications is believed to have broadly increased the number of all genes including K^+^ channel genes (Ohno 1999; Meyer and Van de Peer 2005; Smith et al. 2013).

Evolutionary studies have shown that voltage-gated K^+^ channels are highly conserved in cnidarians and most of the diversification of animal voltage-gated K^+^ channel families and subfamilies predates the radiation of Parahoxozoa (Cnidaria, Placozoa, and Bilateria; Li et al. 2015a, 2015b). Indeed, phylogenetic comparison of voltage-gated K^+^ channel genes found in the sea anemone model organism *Nematostella vectensis* (Anthozoa, starlet sea anemone) to bilaterian channels indicates that eight of the nine predicted bilaterian ancestral channels were also present in the cnidarian/bilaterian ancestor. *Nematostella* contains a single KCNQ paralog that is an outgroup to the two types of bilaterian KCNQs (Li et al. 2015a), but all seven subfamilies of bilaterian Shaker and EAG family channels are conserved in *Nematostella* (Jegla et al. 2012; Li et al. 2015a, 2015b). Furthermore, functional analyses have shown that the biophysical characteristics of voltage-gated K^+^ channel families and subfamilies are also largely conserved between cnidarians and bilaterians (Jegla and Salkoff 1997; Sand et al. 2011; Jegla et al. 1995, 2012; Martinson et al. 2014; Li et al. 2015a, 2015b). Phylogenetic analyses indicate that all *Nematostella* voltage-gated K^+^ channels belong to gene families and subfamilies previously identified in bilaterians and there is no evidence for cnidarian-specific K^+^ channel families or subfamilies (Li et al. 2015a, 2015b).

Given the erroneous assumption that cnidarians are primitively simple animals, it may have been expected that the overall number of K^+^ channels would be similar to the inferred set of nine K^+^ channels in the last common ancestor of Bilateria. However, *Nematostella* harbors 52 voltage-gated K^+^ channel genes and at least 35 exist in the genome of the freshwater hydrozoan *Hydra vulgaris* (Jegla et al. 2012; Li et al. 2015a, 2015b). It is nevertheless unclear how voltage-gated K^+^ channels evolutionarily diversified within Cnidaria and if expanded voltage-gated K^+^ channel sets are a common feature of cnidarian species. Here we compare voltage-gated K^+^ sets across the cnidarian phylogeny to gain insights into patterns of evolutionary diversification and the set of voltage-gated K^+^ channels present in the last common ancestor of cnidarians. We examined eight species (for which there exist high-quality publicly available genomes) whose last common ancestor is believed to be the ancestor of all extant cnidarians (Zapata et al. 2015).

## Results

**Fig. 2.**
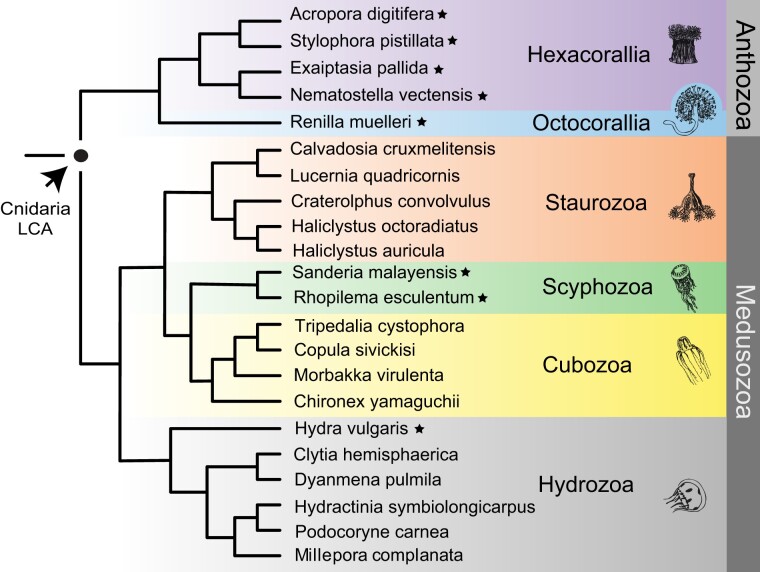
Phylogenetic relationships of cnidarian species. The eight species we comprehensively analyzed for voltage-gated K^+^ channel content (stars) fall into two major lineages (Anthozoa and Medusozoa) whose last common ancestor represents the stem cnidarian lineage. Starred anthozoan species used represent the two anthozoan classes Hexacorallia and Octocorallia, whereas starred medusozoan species used represent two major classes Hydrozoa and Scyphozoa. Additional species included in the tree are represented by sequences in supplemental surveys of voltage-gated K^+^ channel diversity within Octocorallia, Hydrozoa, Staurozoa, and Cubozoa (see supplementary figs. S1–S4, Supplementary Material online). Note no additional octocorallians are included because we did not find evidence additional ancestral voltage-gated K^+^ channels in public transcriptome and genome data beyond what we found in *R. muelleri*.

There is now a wealth of sequence data available from cnidarians (Alama-Bermejo and Holzer 2021; Quek and Huang 2022; Santander et al. 2022), but we chose to limit our main comparisons of voltage-gated K^+^ channels to eight species with high-quality genomes and transcriptomes because estimates of ancestral channel sets depend on having a relatively complete picture of the channel genes present in each species. The species we chose are listed along with a consensus phylogeny of Cnidaria in figure 2 and represent key evolutionary nodes that enable several important comparisons. First, we included both anthozoans and medusozoans which bracket the deepest divergences within Cnidaria; comparison of these groups gives insight into the channel complement present in the last common ancestor of cnidarians. Within the Anthozoa, we used dense sampling of Hexacorallia (*Acropora digitifera*, staghorn coral; *Exaiptasia pallida*, glass anemone; *N. vectensis* and *Stylophora pistillata*, hood coral) to provide a relatively high resolution view of evolutionary events that led to the extensive channel set of the highly developed experimental model *N. vectensis* (Darling et al. 2005). We also included an octocorallian (*Renilla muelleri*, sea pansy) to gain insights into the channel set of the last common anthozoan ancestor. The inclusion of two schyphozoans (*Rhopilema esculentum*, flame jellyfish; *Sanderia malayensis*, pelagic jellyfish) and a hydrozoan (*H. vulgaris*, freshwater hydra) provided us with insight into channel ancestry and diversification within the medusozoan lineage. We did not include Endocnidozoa in our study because they derive from the same common ancestor as Anthozoa and Hydrozoa (Kayal et al. 2018) and have undergone extensive neuronal gene loss (Guo et al. 2022). We therefore reasoned that they would provide limited insight into the voltage-gated K^+^ channel set of stem cnidarians.

We applied a reciprocal best protein BLAST strategy to identify voltage-gated K^+^ channels (see Materials and Methods for details) as had been effectively applied to identify K^+^ and other channels in previous studies (Barzilai et al. 2012; Jegla et al. 2012; Martinson et al. 2014; Baker et al. 2015; Li et al. 2015a, 2015b). On average, we found 44 voltage-gated K^+^ channels in the 8 cnidarian species surveyed, with channel numbers ranging from 36 in *A. digitifera* to 53 in *N. vectensis* (Table 1). This is substantially more than observed in the protostome invertebrate model organisms *Caenorhabditis elegans* (16) and *Drosophila* (9) and similar to vertebrates such as humans (40) (Table 1). Note we found one additional Shaw subfamily K^+^ channel in an updated *N. vectensis* genome draft (Zimmermann et al. 2022) compared with previous studies using the original draft (Putnam et al. 2007). All cnidarian species have extensive Kv families and comparatively smaller KCNQ and EAG families.

**Table 1.** Counts of Voltage-Gated K^+^ Channels in Cnidarians and Select Bilaterians

Gene Family	Kv				EAG			KCNQ
Subfamily	Shaker (Kv1)	Shab (Kv2)	Shaw (Kv3)	Shal (Kv4)	Eag	Erg	Elk	
Cnidaria								
Anthozoa/Hexacorallia								
*Acropora digitifera*	16	0	10	6	1	1	1	1
*Stylophora pistillata*	20	0	11	10	1	4	1	1
*Exaiptasia pallida*	19	1	12	13	1	2	1	0
*Nematostella vectensis*	20	1	12	12	1	5	1	1
Anthozoa/Octocorallia								
*Renilla muelleri*	17	0	10	5	2	2	0	1
Medusozoa/Schyphozoa								
*Rhopilema esculentum*	21	1	7	3	1	2	1	4
*Sanderia malayensis*	22	1	6	3	1	2	1	4
Medusozoa/Hydrozoa								
*Hydra vulgaris*	16	1	15^a^	2	4	4	0	7
Bilateria								
*Homo sapiens*	8	2	4	3	2	3	3	5
*Drosophila melanogaster*	1	1	2	1	1	1	1	1
*Caenorhabditis elegans*	1	6	3	1	1	1	0	3

a Includes an additional gene found in supplemental surveys (supplementary fig. S4, Supplementary Material online).

### Phylogenetic Reconstruction of the KCNQ Family

**Fig. 3.**
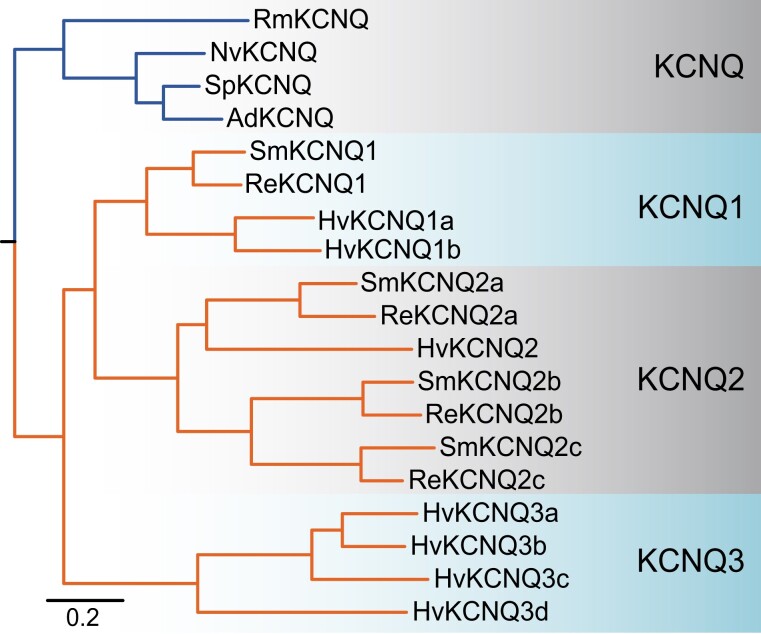
Bayesian inference phylogeny of the cnidarian KCNQ family. Anthozoan sequences are indicated with blue lines and hydrozoan sequences with orange lines. Gene names are given at branch tips. Species prefixes are as follows: Ad, *Acropora digitifera*; Ep, *Exaiptasia pallida*; Hv, *Hydra vulgaris*; Nv, *Nematostella vectensis*; Rm, *Renilla muelleri*; Re, *Rhopilema esculentum*; Sm, *Sanderia malayensis*; Sp, *Stylophora pistillata*. All nodes had a posterior probability ≥0.95 and the scale bar indicates substitutions/site. The phylogeny was unrooted but has a root placed between Anthozoa and Medusozoa for display to represent a single predicted KCNQ channel in the stem cnidarian lineage. Major channel clades described in the results are shaded. Sequences used in the phylogeny are included in supplementary file S1, Supplementary Material online and the tree is included as supplementary File S2, Supplementary Material online.

Li et al. (2015a) identified a single KCNQ channel in three hexacorallian anthozoan genomes (*N. vectensis*, *A. digitifera*, and *Orbicella faveolata*) but six KCNQs in *H. vulgaris*. Here, we identified a seventh KCNQ channel in *H. vulgaris* that was previously missed. Our phylogeny of the KCNQ family is consistent with there being a single KCNQ gene in the last common ancestor of Cnidaria (fig. 3). In addition, there is no evidence of KCNQ channel duplication in the anthozoan lineage. We did not find a complete KCNQ channel in the sea anemone *E. pallida*, but we observed likely KCNQ channel sequence fragments in the genome (data not shown). In contrast, our phylogeny is consistent with a KCNQ duplication in the medusozoan lineage prior to the divergence of scyphozoans and hydrozoans to produce two ortholog groups (KCNQ1 and KCNQ2 in fig. 3). Furthermore, the phylogenetic evidence suggests that KCNQ1 was duplicated in the lineage leading to *Hydra* (KCNQ1a,b), whereas KCNQ2 duplicated twice in the lineage leading to Scyphozoa (KCNQ2a–c). Four additional Hydra KCNQ channels (KCNQ3a–d) form a separate clade in the phylogeny. Unlike all other KCNQ channels, these KCNQ3s share an intron-less gene structure across the conserved core suggesting they are the product of a relatively recent retrotransposition event.

### Phylogenetic Reconstruction of the EAG Family

**Fig. 4.**
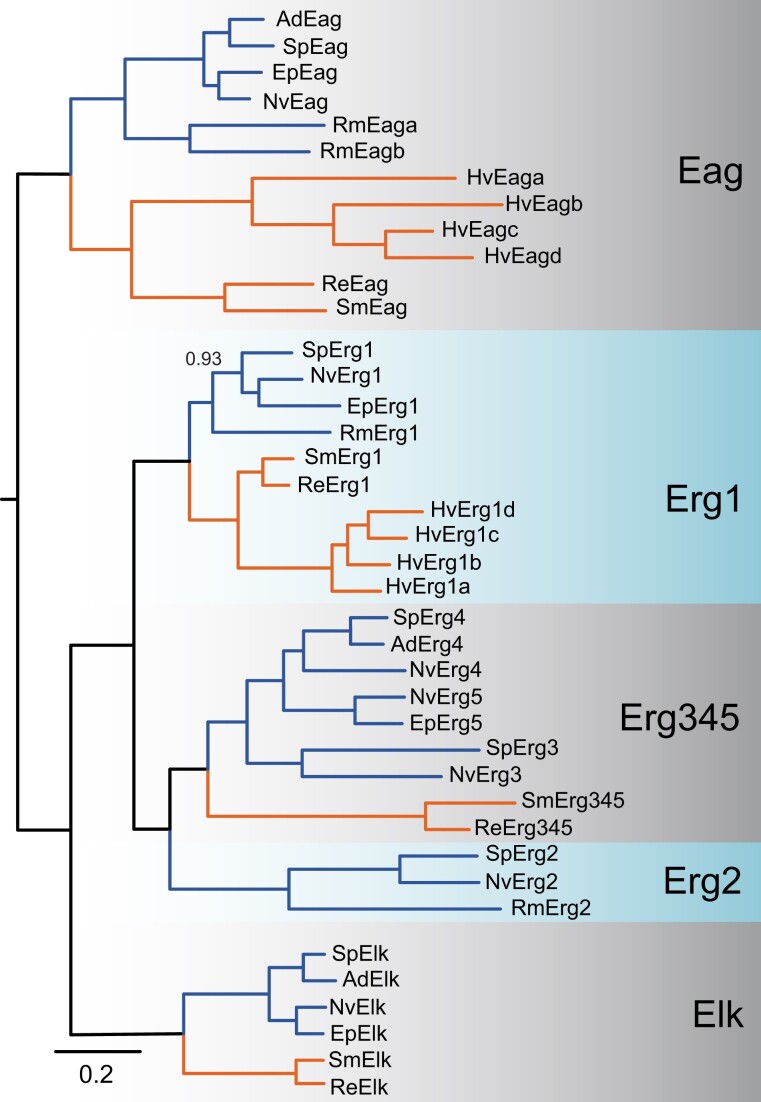
Bayesian phylogeny of the cnidarian EAG family. Anthozoan and medusozoan sequences are indicated with blue and orange lines, respectively. Gene names are given at branch tips. Species prefixes are as follows: Ad, *Acropora digitifera*; Ep, *Exaiptasia pallida*; Hv, *Hydra vulgaris*; Nv, *Nematostella vectensis*; Rm, *Renilla muelleri*; Re, *Rhopilem esculentum*; Sm, *Sanderia malayensis*; Sp, *Stylophora pistillata*. All nodes had a posterior probability ≥0.95 unless otherwise indicated, and the scale bar represents substitutions/site. Ancestral ortholog groups containing both anthozoan and medusozoan sequences and the anthozoan Erg2 clade are shaded. The phylogeny was unrooted but has been rooted between the Eag and Erg/Elk subfamilies for display purposes. Sequences are given in the EAG family section of supplementary file S1, Supplementary Material online and the tree file is supplementary file S3, Supplementary Material online.

Our phylogenetic analyses of cnidarian EAG family channels suggest that the last common cnidarian ancestor had four EAG family channels: a single gene for the Eag and Elk gene subfamilies and two genes for the Erg subfamily (fig. 4). The EAG family alignment was limited to the S1–S6 transmembrane channel core, the C-linker and the CNBHD, as not all cnidarian Erg subfamily channels contain an N-terminal eag gating domain (Martinson et al. 2014). The single cnidarian Eag ancestor was separately duplicated once in the *Re. muelleri* lineage and three times in the *H. vulgaris* lineage (fig. 4). We found no evidence for duplication of the single Elk subfamily ancestor. We did not find an Elk channel in *Re. muelleri* or *H. vulgaris* (fig. 4), raising the possibility of Elk gene loss, as has been established for *C. elegans* in Bilateria (Jegla et al. 2009; Li et al. 2015b).

Previous phylogenetic and functional analysis of *Nematostella* Erg subfamily revealed five total Erg genes falling into two clades differentiated by the presence (NvErg1) or absence (Nverg2-5) of the eag gating domain (Martinson et al. 2014). NvErg1 is closely related to bilaterian Erg channels and encodes a channel with high functional similarity to human cardiac Erg channels (Martinson et al. 2014). The NvErg2-5 clade had only been reported from *Nematostella* and NvErg4 is functionally similar to Erg channels from fly and worm, which independently lost the eag gating domain (Martinson et al. 2014). Here, we show that both of these *Nematostella* Erg clades were present in the last common ancestor of Cnidaria (fig. 4). The Erg1 ancestor has been duplicated three times specifically in *Hydra*, but these *Hydra* Erg1-like channels lack the eag gating domain and thus appear to represent a second evolutionarily independent loss of the eag gating domain in cnidarian Erg channels. Duplications within the NvErg2–5 group were restricted to the anthozoans, with 1–4 genes present in each species we examined. Within anthozoans, we found evidence for an Erg2 ortholog in Octocorallia and Hexacorallia, whereas an Erg345 clade was restricted to subsets of the Hexacorallia (fig. 4). The Erg2-like and Erg345-like channels were not detected in Hydra, but we did find an Erg345 ortholog in scyphozoans. Thus, it appears an Erg2/Erg345 ancestor was present in the last common cnidarian ancestor, but the channel lineage may have been lost in *H. vulgaris*.

### Phylogenetic Reconstruction of the Kv Family

**Fig. 5.**
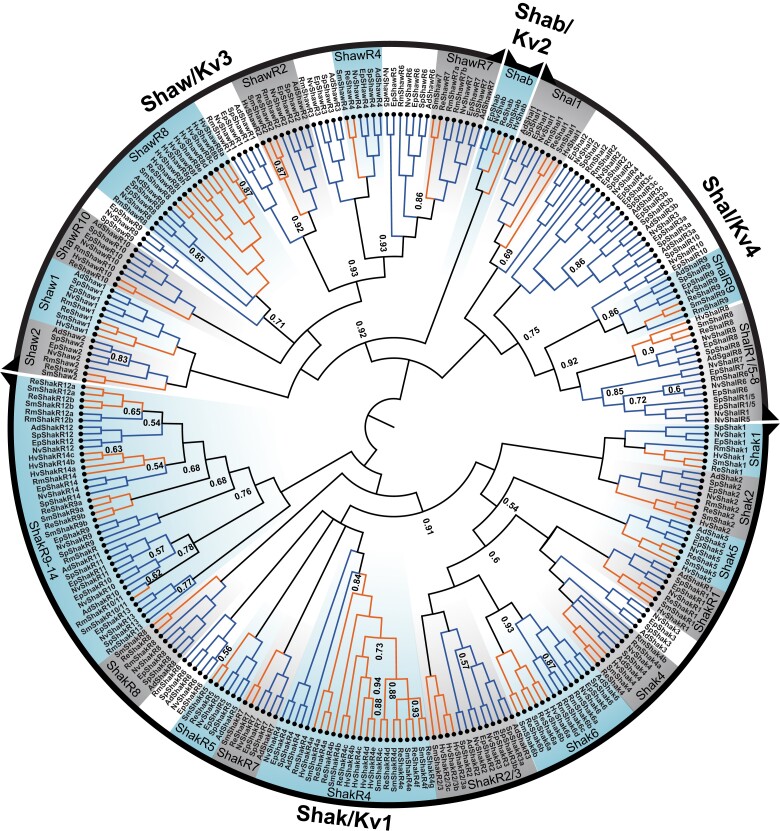
Bayesian inference phylogeny of the cnidarian Kv family. The Shaker (Kv1), Shab (Kv2), Shaw (Kv3), and Sha1 (K4) subfamilies are labeled at the perimeter and the unrooted phylogeny is rooted between Shaker and Shab/Shal/Shaw to reflect the earlier origin of the Shaker subfamily (Li et al. 2015a). Anthozoan and hydrozoan sequences are indicated with blue and orange lines, respectively, and nodes were supported at >0.95 posterior probability unless otherwise labeled. Gene names are given at branch tips. Species prefixes are as follows: Ad, *Acropora digitifera*; Ep, *Exaiptasia pallida*; Hv, *Hydra vulgaris*; Nv, *Nematostella vectensis*; Rm, *Renilla muelleri*; Re, *Rhopilem esculentum*; Sm, *Sanderia malayensis*; Sp, *Stylophora pistillata*. Branch lengths are not shown for display purposes but are included in the tree file (supplementary file S4, Supplementary Material online). Ancestral ortholog groups containing anthozoan and medusozoan sequences are shaded and labeled. Sequences are given in the Kv family table within supplementary file S1, Supplementary Material online.

We found 23 Kv family clades containing both anthozoan and medusozoan sequences, providing support a total of at least 23 Kv family channels present in the last common ancestor of cnidarians (fig. 5). Twelve of these ancestral anthozoan/medusozoan ortholog groups were found in the Shaker subfamily, one in the Shab subfamily, seven in the Shaw subfamily and three in the Shal subfamily (fig. 5). We recovered 21 anthozoan/medusozoan clades with maximum clade support (posterior probability of 1), and 2 additional clades with posterior probabilities >0.93 (fig. 5). We treat a large clade represented by NvShakR9-14 as a single ancestral group; although it has multiple subclades containing both anthozoans and medusozoans, statistical support for these subclades was weak (fig. 5). The 45-gene *Nematostella* Kv family is rounded out by 22 additional gene duplications, including 7 in the anthozoan ancestor (present in both Octocorallia and Hexacorallia), 5 within Hexacorallia (present in corals and anemones), 4 in anemones (present in *Nematostella* and *Exaiptasia*), and only 1 in the *Nematostella* lineage after it diverged from *Exaiptasia* (fig. 5). The timing of the gene duplications producing NvShakR9-14 is unclear due to low phylogenetic support, but the phylogeny supports the presence of all six genes in the hexacorallian ancestor.

**Fig. 6.**
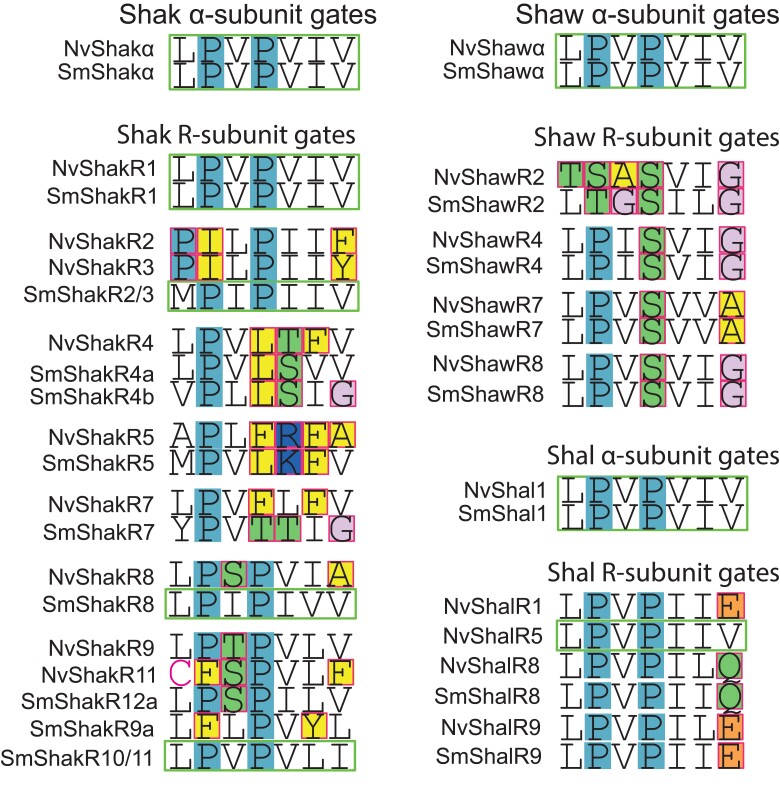
Comparison of S6 activation gate sequences for Kv α-subunits and R-subunits. Shaker subfamily examples are shown in the left column and Shaw and Shal subfamily examples are shown in the right column. For each subfamily, consensus α-subunit activation gate sequences (green outline) are shown above examples of sequences from ortholog groups containing *Nematostella* R-subunits. R-subunit gate positions with nonconservative mutations (in terms of gate structure) are outlined in red and highlighted based on chemical properties, and α-like R-subunit gates with no unusual substitutions are outlined in green. Example sequences are included for *N. vectensis* and *S. malayensis* orthologs to illustrate whether gate changes appear before or after divergence of the anthozoans and medusozoans. In R-subunit clades where gate changes appear ancestral (found in both anthozoans and medusozoans), individual sequences with normal α-like gates (outlined in green) could represent reversion mutations. In R-subunit clades where gate mutations occur in both *Nematostella* and *Sanderia*, the changes are also found in the other anthozoan and medusozoan species represented in the study (see supplementary file S1, Supplementary Material online).

Kv channel subunits can be functionally divided into α-subunits that have the capacity to form functional homotetrameric channels and “silent” or “regulatory” subunits, referred to as R-subunits here, that require heteromeric assembly with α-subunits for function (Pisupati et al. 2018). Kv R-subunits have evolved independently multiple times in animals (Pisupati et al. 2018). They have been identified by functional analysis in the Shaker, Shaw, and Shal subfamilies of *Nematostella* (Jegla et al. 2012; Li et al. 2015a), and the Shal subfamily of the hydrozoan *Polyorchis penicillatus* (Jegla and Salkoff 1997). In our 8 species cnidarian Kv phylogeny, 9 of the ancestral cnidarian Kv clades contain *Nematostella* α-subunits and the remaining 14 contain only *Nematostella* R-subunits. One unusual sequence feature of R-subunits is degeneration of the remarkably conserved S6 activation gate consensus sequence of α-subunits; this has been hypothesized to occur selectively in R-subunits because they can accumulate mutations that can be tolerated in only 1–2 subunits of a functional tetramer (Pisupati et al. 2018). Indeed, nonconservative gate changes are present in 43 of 44 *Nematostella* and mouse R-subunits (Pisupati et al. 2018), with only NvShakR1 having a normal α-like gate sequence. Both *Sanderia* and *Nematostella* orthologs showed alterations to the gate sequence in 11 of 14 R-subunit ortholog groups (fig. 6), suggesting that the R-assembly phenotype might have been present in these channels prior to the divergence of anthozoans and medusozoans. ShakR1 did not show changes in anthozoans or medusozoans, whereas the ShakR8 and ShakR2/R3 only had altered gates in anthozoans.

### The Voltage-Gated K^+^ Channel Gene Set of Stem Cnidarians

**Fig. 7.**
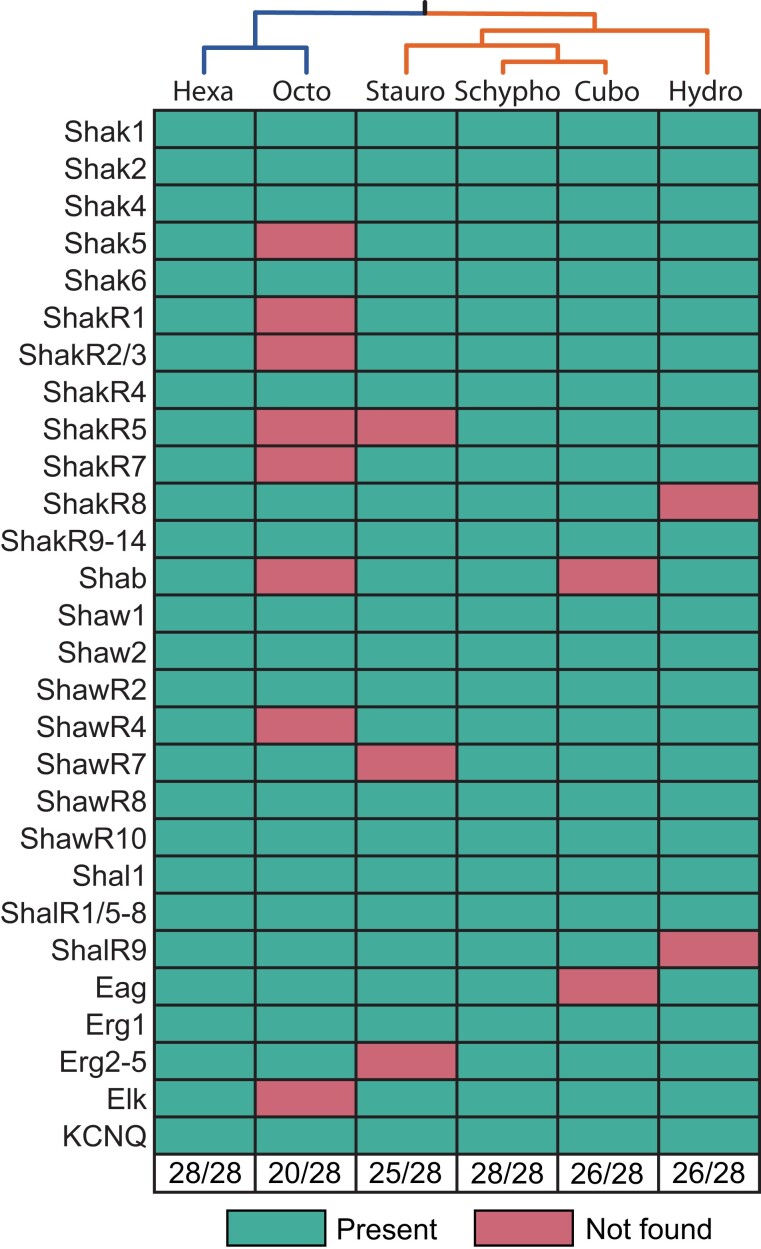
Conservation of ancestral voltage-gated K^+^ channels across Cnidaria. The chart shows the presence (green) or absence (red) of 28 voltage-gated K^+^ channel genes predicted to have been present in stem cnidarians for each of 6 cnidarian classes. Relationships between the classes are indicated by the tree at the top margin. Absent genes may have been lost in the indicated lineages.

Our cnidarian Kv, KCNQ and EAG families (figs. 3–5) support a total of 28 voltage-gated potassium channel genes in the last common ancestor of cnidarians (fig. 7). We found all 28 channels in Hexacorallia and Scyphozoa, but only 19 in Hydrozoa and Octocorallia. Because *H. vulgaris* and *Re. muelleri* were respectively the sole representatives of these classes in our initial analyses, we were concerned that the apparent low retention of ancestral voltage-gated K^+^ channels might be due to taxon sampling rather than gene loss. We therefore performed searches of publicly available cnidarian transcriptome and genome data from additional species (1) to look for orthologs of missing ancestral channels in Hydrozoa and Octocorallia, and (2) to get a preliminary read on voltage-gated K^+^ channel diversity in two additional medusozoan classes, Staurozoa and Cubozoa. Note this broader supplementary search was designed simply to look for the presence of ancestral voltage-gated K^+^ channel in various cnidarian classes and was not intended to provide insight into the full channel complement of additional species. Kv, KCNQ, and Eag phylogenies containing voltage-gated K^+^ channel sequences from additional cnidarian species (listed in fig. 2) are shown in supplementary figures S1–S4, Supplementary Material online, the additional sequences are included in supplementary file S1, Supplementary Material online and tree files are included as supplementary files S5–S7, Supplementary Material online. This survey yielded evidence for conservation of seven additional ancestral cnidarian voltage-gated K^+^ channels in Hydrozoa (including a *H. vulgaris* ShawR4 ortholog that we had previously missed), but no evidence for additional ancestral sequences in Octocorallia (fig. 7). We also found evidence for 25 of 28 ancestral cnidarian voltage-gated K^+^ channels in Staurozoa and 26 of 28 in Cubozoa (fig. 7). Thus, among the cnidarian classes we examined, only Octocorallia has lost a significant fraction of the striking diversity of voltage-gated K^+^ channels inherited from stem cnidarians. However, it remains possible that some of these predicted losses are artifacts of incomplete transcriptome coverage or even fast evolving channels that are no longer recognizable by BLAST (Martín-Durán et al. 2017), and they will need to be revisited as additional sequence data become available.

## Discussion

The most striking finding in our study is that the diversity of voltage-gated K^+^ channels in the last common ancestor of cnidarians was >3× higher than that of the last common ancestor of bilaterians. Although alternative splicing has been demonstrated to play a role in diversifying a few bilaterian voltage-gated K^+^ channels such as Shaker (Kv1) in *D. melanogaster* (Kamb et al. 1988; Timpe et al. 1988), this alternative splicing is not conserved across Bilateria (Jegla et al. 2012) and therefore, there is no evidence that it contributed significantly to the diversity of voltage-gated K^+^ channels in ancestral bilaterians. We did not examine alternative splicing in detail for this study, but there is little evidence for splicing within the conserved core domains of *Nematostella* Kv family channels (Jegla et al. 2012). As such, the large number of voltage-gated K^+^ channel genes we predict here for stem cnidarians most likely represents a real increase in voltage-gated K^+^ channel diversity within the stem cnidarian lineage compared with the stem bilaterian lineage. How ancestral cnidarians may have deployed such a large set of voltage-gated K^+^ channels is not clear given our current rudimentary understanding of cnidarian electrical signaling and cellular communication. In addition to regulating excitability, it is likely that at least a few of the channels are dedicated to regulating the physiology of nonexcitable cells as has been observed in vertebrates. We did not examine the diversity and conservation of other types of K^+^ channels across Cnidaria in this study, but *N. vectensis* has at least 36 additional K^+^ channel genes collectively in the Kir, K2P, SK and BK gene families which encode, inward rectifier, voltage-independent, Ca^2+^-activated and Na^+^-activated K^+^ channels (Jegla et al. 2009). This and the observed functional conservation of cnidarian voltage-gated K^+^ channels (Jegla et al. 2012; Li et al. 2015a, 2015b) suggest that the diversification of cnidarian voltage-gated K^+^ channels does not compensate for a deficit in other K^+^ channel types. The conservation of voltage-gated K^+^ channel diversity over long evolutionary times in all cnidarian lineages therefore suggests a functionally diverse set of voltage-gated K^+^ channels is a central requirement and feature of cnidarian physiology. Excitingly, this extensive functional diversification of cnidarian K^+^ channels presents an opportunity from which to understand cnidarian physiology through functional analyses.

Broad in vitro functional characterization of *Nematostella* voltage-gated K^+^ channels has demonstrated a high level of diversity in gating properties beyond what is typical for bilaterian voltage-gated K^+^ channels (Jegla et al. 2012; Li et al. 2015a, 2015b). This suggests that excitable cells in cnidarians are electrically complex and highly diverse, but there is yet very little information on whether these gating properties are highly conserved across Cnidaria. There are only three published examples of functional comparisons of anthozoan (*Nematostella*) and medusozoan (hydrozoan) voltage-gated K^+^ channel orthologs. NvShak1 (Jegla et al. 2012) and its orthologs from the hydrozoan jellyfish *Po. penicillatus* (Jegla et al. 1995) and *Physalia physalis* (Bouchard et al. 2006) have similar high activation thresholds and share classical fast N-type inactivation that was first described for *Drosophila* Shaker (Hoshi et al. 1990; Zagotta et al. 1990). Similarly, *N. vectensis* NvShaw1 (Li et al. 2015a) shares an atypical low activation threshold with jShaw1, a hydrozoan ortholog from *Po. penicillatus* (Sand et al. 2011). In contrast, the gating properties of NvShal1 (Li et al. 2015a) and its *Polyorchis* ortholog jShal1 have large differences in both voltage-dependence and kinetics (Jegla and Salkoff 1997). More extensive functional comparisons of orthologous K^+^ channels across Cnidaria are needed to understand to what extent gating properties of the large cnidarian voltage-gated K^+^ channel set have been conserved. The sequences and phylogenies described here will help guide future functional comparisons.

These large compliments of voltage-gated K^+^ channels almost certainly also provide cnidarians with diversity in cell-type specificity, subcellular localization specificity, and association with distinct macromolecular signaling complexes. Nevertheless, very little is understood regarding the functions of these K^+^ channels in vivo, and even less is known about functional differences between cnidarian species. *Nematostella* Shakers do have diverse expression patterns as measured by in situ hybridization in juvenile polyps (Jegla et al. 2012). NvShak1 appears to be expressed in tentacles (Jegla et al. 2012) where cnidocytes are concentrated, and its *Ph. physalis* ortholog was cloned from cnidocytes (Bouchard et al. 2006). However, there is no other comparative information on the expression patterns of cnidarian voltage-gated K^+^ channels. Single-cell transcriptomes have been useful for defining cell-type diversity in cnidarians (Sebe-Pedros et al. 2018; Siebert et al. 2019), but have not yet been sequenced in sufficient depth to provide useful information on cell-type specific expression of ion channels, which are typically low abundance transcripts. Continued development of cnidarians as model organisms especially for neuroscience will be needed to shed light on the role of high channel diversity in cnidarian physiology.

Our results show ancestral diversity both in standard Kv α-subunits and Kv R-subunits. Although we cannot be certain that the requirement for heteromeric assembly observed functionally for *Nematostella* R-subunits (Jegla et al. 2012) is conserved in medusozoan orthologs without functional experiments, the degeneration of the activation gate sequence in both anthozoans and medusozoans in 11 putative R-subunit clades is highly suggestive because gate sequence degeneration is not observed in Kv α-subunits (Pisupati et al. 2018). However, it should be noted that activation gate changes in mammalian Shab subfamily R-subunits are a marker for the R-assembly phenotype due to relaxed selection but are not causal for the R-subunit assembly phenotype (Pisupati et al. 2018). Thus, there is no requirement for gate changes in R-subunits and presumably no barrier to reversion to an α-like gate. This could explain the presence of sporadic sequences with α-like gates in some putative cnidarian R-subunit clades (fig. 6) or the α-like gate in ShakR1 orthologs (Jegla et al. 2012; Pisupati et al. 2018). The potential conservation of both α-subunits and R-subunits throughout Cnidaria is interesting because it suggests that specific types of heteromeric Kv channels have been selected and conserved. Detailed examination of gene expression patterns in *Nematostella* coupled with functional expression experiments could reveal these ancestral heteromeric Kv combinations.

One interesting question that arises given the unexpectedly high ancestral cnidarian voltage-gated K^+^ channel diversity is which channels were present in the last common cnidarian/bilaterian common ancestor? The simplest explanation of our new data and previous publications is that cnidarian voltage-gated K^+^ channels diversified specifically within the cnidarian stem lineage after the divergence of cnidarians and bilaterians but before the diversification of the major cnidarian lineages. In this case, we would predict a last common cnidarian/bilaterian ancestor with a simple set of eight voltage-gated K^+^ channels, one each of Shaker, Shab, Shal, Shaw, KCNQ, Eag, Erg, and Elk. The alternative hypothesis that at least some of the excess voltage-gated K^+^ channel diversity we predict for stem cnidarians was already present in the last common ancestor of cnidarians and bilaterians and subsequently lost in the bilaterian lineage is less parsimonious. We favor the first hypothesis because (1) cnidarian and bilaterian channels branch separately in phylogenies (Jegla et al. 2012; Martinson et al. 2014; Li et al. 2015a, 2015b), (2) *Trichoplax*, the sister lineage to cnidarians and bilaterians, shares none of the cnidarian duplications (Martinson et al. 2014; Li et al. 2015a, 2015b), and (3) comparison with the earlier branching ctenophore lineage suggests that the diversification of these channels within Ctenophora was independent and only two voltage-gated K^+^ channel genes were present in the ctenophore/cnidarian/bilaterian ancestor (Li et al. 2015a, 2015b).

In summary, we find large, sequence-diverse sets of voltage-gated K^+^ channels in all free-living cnidarians. Much of this diversity can be traced to the cnidarian common ancestor; there appears to have been a burst of K^+^ channel diversification in the stem cnidarian lineage prior to emergence of the extant classes. There have also been numerous lineage-specific gene duplications and some putative lineage-specific gene losses that differentiate the voltage-gated K^+^ channel sets of the major cnidarian lineages. Understanding the evolution of K^+^ channels in a wide range of cnidarian lineages is an important step toward understanding the evolution of the excitable cells and networks that led to the establishment of Cnidaria. These data help set the stage for subsequent functional work to deepen our understanding of the physiology of these surprisingly complex animals.

## Materials and Methods

### Sequence Searches

We obtained Kv, EAG, and KCNQ family protein sequences through BLAST searches using all identified *N. vectensis* (Cnidaria, Anthozoa, and Hexacorallia) Kv, EAG, and KCNQ sequences as queries (Li et al. 2015a, 2015b). We performed BLASTP searches against protein model databases from the following cnidarian species for which genomes were available: *St. pistillata* (Anthozoa, Hexacorallia [Voolstra et al. 2017]), *A. digitifera* (Anthozoa, Hexacorallia [Shinzato et al. 2011]), *E. pallida* (Anthozoa, Hexacorallia [Baumgarten et al. 2015])*, Re. muelleri* (Anthozoa, Octocorallia [Jiang et al. 2019]), *H. vulgaris* (Hydrozoa [Chapman et al. 2010]), *Sa. malayensis* (Scyphozoa [Nong et al. 2020]), *Rh. esculentum* (Scyphozoa [Nong et al. 2020]). We designated as complete and appropriate for phylogenetic analysis, those channel sequences that had the following: the voltage-gated K^+^ channel core motifs (S1–S6) plus the T1 domain for Kvs, the coiled-coil C-terminal tail for KCNQ, or the EAG/PAS and CNBD domains in EAG channels (excluding Erg subfamily genes lacking the EAG domain and certain *H. vulgaris* Eag sequences). We used “complete” Kv sequences as queries for reciprocal best BLASTP searches against the *N. vectensis* protein database and used “complete” EAG and KCNQ sequences as queries for reciprocal best BLASTP searches against *Mus musculus* Refseq (O'Leary et al. 2016). Hits with a best reciprocal match to the family/subfamily of the original query and an *E*-value <0.01 were used in further analyses.

We generated alignments for our main phylogenetic analyses using MUSCLE (Edgar 2004) as implemented in MEGA X (Kumar et al. 2018). We removed surrounding unconserved regions from the resulting alignments, leaving only alignments of conserved domains. Some predicted sequences had gaps in the conserved domains identified via alignment. We conducted TBLASTN searches using orthologs from other cnidarian species as queries against respective genome and/or transcriptome data and, in some cases, were able to fill in residues that were missing from gene models. In cases where we identified potentially missing residues, we only included these data if we were able to confirm the following: that these sequences were on the same genomic contig as the original sequence, were in the same orientation as the original sequence, and that predicted exons included putative splice sites capable of inserting the exon into the predicted sequence in the proper position in frame. We removed from subsequent analyses several sequences that were still missing >10% of the aligned region. Total alignment lengths were 280 amino acids for the shaker family, 427 amino acids for the EAG family, and 345 amino acids for the KCNQ family. A list of full channel and alignment sequences can be found in supplementary file S1, Supplementary Material online.

### Phylogenetic Analysis

We constructed individual phylogenies for each gene family via Bayesian inference using MrBayes (v3.2.7a) (Ronquist et al. 2012) with BEAGLE 3 (Ayres et al. 2019) by Markov Chain Monte Carlo sampling for 1,000,000 generations (6 simultaneous chains, sampled every 5,000 generations, mixed amino acid model). All trees converged with a standard deviation of split frequencies <0.01. To identify ancestral pan-cnidarian voltage-gated K^+^ channel genes, we identified the smallest clades containing genes from at least one anthozoan and medusozoan species in each of the three phylogenies. We only considered clades with posterior probabilities>0.95, excepting the NvShak6 clade, which had a posterior probability of 0.93. All sequences used in the phylogenies are provided in supplementary file S1, Supplementary Material online, and the tree files for the KCNQ, EAG, and Kv phylogenies are provided as supplementary files S2–S4, Supplementary Material online.

## Supplementary Material

evad009_Supplementary_Data

## Data Availability

The analyses presented here were based on sequence data in GenBank. Specific details on sequences used are provided in the Supplementary material.
