# Supplementary material for: Genome-Scale Analysis Reveals Extensive Diversification of Voltage-Gated K+ Channels in Stem Cnidarians
Source: Genome Biol Evol. 2023 Jan 21;15(3):evad009. doi: 10.1093/gbe/evad009 (PMC9989356; doi:10.1093/gbe/evad009)
Supplement: evad009_Supplementary_Data [file evad009_supplementary_data.zip › Supplemental text and figures.pdf]

Supplemental methods

Expanded KCNQ and EAG sequence searches. We searched transcriptomes shotgun assembly databases at NCBI for Cubozoa, Staurozoa and Hydrozoa for EAG and KCNQ channels with TBLASTN using *Nematostella vectensis* gene family members as queries. Channel sequences were considered complete if they contained the voltage-gated K^+ channel core motifs (S1-S6) plus the coiled-coil C-terminal tail for KCNQ or the EAG/PAS and CNBD domains for EAG. Retrieved sequences were translated and used as queries in reciprocal BLASTP searches against mouse Refseq (O'Leary, Wright et al. 2016) to confirm gene family/subfamily identity.

Sequences for the following 8 representative species were added to the multiple sequence alignments used for Fig. 3 and 4 using the same alignment methods: *Chironex yamaguchi* (Cubozoa) (Khalturin, Shinzato et al. 2019), *Copula sivickisi* (Cubozoa) (Khalturin, Shinzato et al. 2019), *Calvadosia cruxmelitensis* (Staurozoa) (Kayal, Bentlage et al. 2018), *Lucernaria quadricornis* (Staurozoa) (Kayal, Bentlage et al. 2018), *Clytia haemispherica* (Hydrozoa) (Leclère, Horin et al. 2019), *Dynamena pumila* (Hydrozoa) (Kupaeva, Konorov et al. 2020), *Podocoryne carnea* (Hydrozoa) (Sanders and Cartwright 2015), *Hydractinia symbiolongicarpus* (Hydrozoa) (Sanders and Cartwright 2015). Total alignment lengths 427 amino acids for the EAG family and 345 amino acids for the KCNQ family. The full sequences and aligned sequences can be found in EAG and KCNQ tables in File S1.

Expanded Kv sequence searches. We searched for additional Kv sequences in transcriptome shotgun assembly databases at NCBI for Octocorallia, Cubozoa, Staurozoa, and Hydrozoa. For Octocorallia, we used all *N. vectensis* sequences as TBLASTN search queries, and we used scyphozoan sequences from each clade found in Fig. 5 as search queries for each of the

remaining classes. For the top hits in each query, we performed a reciprocal best BLASTP search against the respective query species. Up to two complete sequences from unique species that had a best match to the original query were used in phylogenetic analyses. Where there were no best matches or incomplete sequences, we searched whole-genome shotgun contigs on NCBI to find whole sequences or fill in gaps from transcriptome data. Sequences for the following 12 species were added to the multiple sequence alignment used for Fig. 5 following the same alignment methods: *Chironex yamaguchi* (Cubozoa) (Khalturin, Shinzato et al. 2019), *Copula sivickisi* (Cubozoa) (Khalturin, Shinzato et al. 2019), *Morbakka virulenta* (Cubozoa) (Khalturin, Shinzato et al. 2019), *Tripedalia cystophora* (Cubozoa) (Khalturin, Shinzato et al. 2019), *Calvadosia cruxmelitensis* (Staurozoa) (Kayal, Bentlage et al. 2018), *Lucernaria quadricornis* (Staurozoa) (Kayal, Bentlage et al. 2018), *Haliclystus auricula* (Staurozoa) (Kayal, Bentlage et al. 2018), *Haliclystus octoradiatus* (Staurozoa) (WGS: CAKAJJ000000000) , *Craterolophus convolvulus* (Staurozoa) (Kayal, Bentlage et al. 2018), *Clytia haemispherica* (Hydrozoa) (Leclère, Horin et al. 2019), *Hydractinia symbiolongicarpus* (Hydrozoa) (Sanders and Cartwright 2015), *Millepora complanata* (Hydrozoa) (Hernández-Elizárraga, Olguín-López et al. 2021). Full and aligned sequences can be found in the Kv table in File S1. Total alignment length was 280 amino acids.

Expanded phylogenetic analysis. Phylogenetic analyses for the EAG, KCNQ, and Kv family trees with the additional transcriptome and high-throughput genomic data were performed in the same manner as in Figs. 3-5. Phylogenies were contructed via Bayesian inference using MrBayes (v3.2.7a) (Ronquist, Teslenko et al. 2012) with BEAGLE 3 (Ayres, Cummings et al. 2019) by Markov Chain Monte Carlo (MCMC) sampling for 1,000,000 generations for the EAG and KCNQ families, and 4,000,000 generations for the Kv family (six simultaneous chains, sampled

every 5000 generations, mixed amino acid model). All trees converged with a standard deviation of split frequencies <0.01. We identified the smallest clades containing genes from anthozoans and medusozoan with posterior probabilities <0.95 as pan-cnidarian (except for ShawR4 which had a posterior probability of 0.93 in the supplemental Kv analysis only). Note Shak6 which had a posterior probability of 0.93 in Fig. 5 had consensus support in this expanded analysis. Tree files for the KCNQ, EAG and Kv phylogenies are provided as Files S2-S4.

Supplemental References

Ayres, D. L., et al. (2019). "BEAGLE 3: Improved Performance, Scaling, and Usability for a High-Performance Computing Library for Statistical Phylogenetics." Syst Biol **68**(6): 1052-1061.

Hernández-Elizárraga, V. H., et al. (2021). "Comprehensive Metatranscriptome Analysis of the Reef-Building Holobiont Millepora complanata." Frontiers in Marine Science **8**.

Kayal, E., et al. (2018). "Phylogenomics provides a robust topology of the major cnidarian lineages and insights on the origins of key organismal traits." BMC evolutionary biology **18**(1): 68-18.

Khalturin, K., et al. (2019). "Medusozoan genomes inform the evolution of the jellyfish body plan." Nature Ecology & Evolution **3**(5): 811-822.

Kupaeva, D., et al. (2020). "De novo transcriptome sequencing of the thecate colonial hydrozoan, Dynamena pumila." Marine genomics **51**.

Leclère, L., et al. (2019). "The genome of the jellyfish Clytia hemisphaerica and the evolution of the cnidarian life-cycle." Nature Ecology & Evolution **3**(5): 801-810.

O'Leary, N. A., et al. (2016). "Reference sequence (RefSeq) database at NCBI: Current status, taxonomic expansion, and functional annotation." Nucleic acids research **44**(1): D733-D745.

Ronquist, F., et al. (2012). "MrBayes 3.2: Efficient Bayesian Phylogenetic Inference and Model Choice Across a Large Model Space." Systematic biology **61**(3): 539-542.

Sanders, S. M. and P. Cartwright (2015). "Interspecific differential expression analysis of RNA-Seq data yields insight into life cycle variation in hydractiniid hydrozoans." Genome biology and evolution **7**(8): 2417-2431.

Supplemental Figure Legends

Figure S1. Expanded Bayesian inference phylogeny of the cnidarian KCNQ family. Anthozoan sequences are indicated with blue lines and medusozoan sequences with orange lines. Gene names are given at branch tips. Species prefixes are as follows: Ad, *Acropora digitifera*; Cc, *Calvadosia cruxmelitensis*; Cy, *Chironex yamaguchii*; Ch, *Clytia hemisphaerica*; Cs, *Copula* sivickisi; Dp, *Dynamena pulmila*; Ep, *Exaptasia pallida*; Hs, *Hydractinia symbiolongicarpus*; Hv, Hydra vulgaris; Lq, *Lucernaria quadricornis*; Nv, *Nematostella vectensis*; Pc, *Podocoryne carnea*; Rm, *Renilla muelleri*; Re, *Rhopilema esculentum*; Sm, *Sanderia malayensis*; Sp, *Stylophora pistillata*. Posterior probabilities for nodes were > 0.95 unless otherwise indicated. The phylogeny was unrooted and has a root was placed between anthozoan and medusozoan sequences for display purposes. Sequences are provided in File S1 and the tree file is provided as File S5.

Figure S2. Expanded Bayesian inference phylogeny of the cnidarian EAG family. Anthozoan sequences are indicated with blue lines and medusozoan sequences with orange lines. Gene names are given at branch tips. Species prefixes are as follows: Ad, *Acropora digitifera*; Cc, *Calvadosia cruxmelitensis*; Cy, *Chironex yamaguchii*; Ch, *Clytia hemisphaerica*; Cs, *Copula* sivickisi; Dp, *Dynamena pulmila*; Ep, *Exaptasia pallida*; Hs, *Hydractinia symbiolongicarpus*; Hv, Hydra vulgaris; Lq, *Lucernaria quadricornis*; Nv, *Nematostella vectensis*; Pc, *Podocoryne carnea*; Rm, *Renilla muelleri*; Re, *Rhopilema esculentum*; Sm, *Sanderia malayensis*; Sp, *Stylophora pistillata*. Posterior probabilities for nodes were >0.95 unless labeled and the unrooted phylogeny is shown with a root between the Erg and Eag/Elk subfamilies for display

purposes. Ancestral ortholog groups containing anthozoan and medusozoan sequences are shaded. Sequences are given in the EAG tables in File S1 and the full tree file is provided as File S6.

Figure S3. Expanded phylogeny of the Kv family I: the Shaker subfamily. The Shaker (Kv1) subfamily is displayed in full, and the Shab (Kv2), Shaw (Kv3), and Shal (Kv4) subfamilies are collapsed for display purposes. Anthozoan and medusozoan lineages are indicated by blue and orange lines, respectively and ancestral ortholog groups are shaded. Nodes were supported at >0.95 posterior probability unless otherwise labeled. Gene names are given at branch tips and species prefixes are as follows: Ad, *Acropora digitifera*; Cc, *Calvadosia cruxmelitensis*; Cy, *Chironex yamaguchii*; Ch, *Clytia hemisphaerica*; Ccon, Craterolphus convolvulus; Cs, *Copula* sivickisi; Ep, *Exaptasia pallida*; Ha, *Haliclystus auricula*; Ho, *Haliclystus octoradiatus*; Hs, *Hydractinia symbiolongicarpus*; Hv, Hydra vulgaris; Lq, *Lucernaria quadricornis*; Mc, Millepora complanate; Mv, *Morbakka virulenta*; Nv, *Nematostella vectensis*; Rm, *Renilla muelleri*; Re, *Rhopilema esculentum*; Sm, *Sanderia malayensis*; Sp, *Stylophora pistillata*; Tc, Tripedalia cystophora. Sequences are provided in the Kv family table withing File S1, and the full expanded Kv family tree is File S7.

Figure S4. Expanded phylogeny of the Kv family II: the Shab, Shal and Shaw subfamilies. The Shab (Kv2), Shaw (Kv3), and Shal (Kv4) subfamilies are displayed in full, and the Shaker (Kv1) subfamily is collapsed for display purposes. Anthozoan and medusozoan sequences are indicated by blue and orange lines, respectively. Nodes support is >0.95 posterior probability unless otherwise indicated. Gene names are given at branch tips with species prefixes as follows:

Ad, *Acropora digitifera*; Cc, *Calvadosia cruxmelitensis*; Cy, *Chironex yamaguchii*; Ch, *Clytia hemisphaerica*; Ccon, Craterolphus convolvulus; Cs, *Copula* sivickisi; Ep, *Exaptasia pallida*; Ha, *Haliclystus auricula*; Ho, *Haliclystus octoradiatus*; Hs, *Hydractinia symbiolongicarpus*; Hv, Hydra vulgaris; Lq, *Lucernaria quadricornis*; Mc, Millepora complanate; Mv, *Morbakka virulenta*; Nv, *Nematostella vectensis*; Rm, *Renilla muelleri*; Re, *Rhopilema esculentum*; Sm, *Sanderia malayensis*; Sp, *Stylophora pistillata*; Tc, Tripedalia cystophora.

Fig. S1

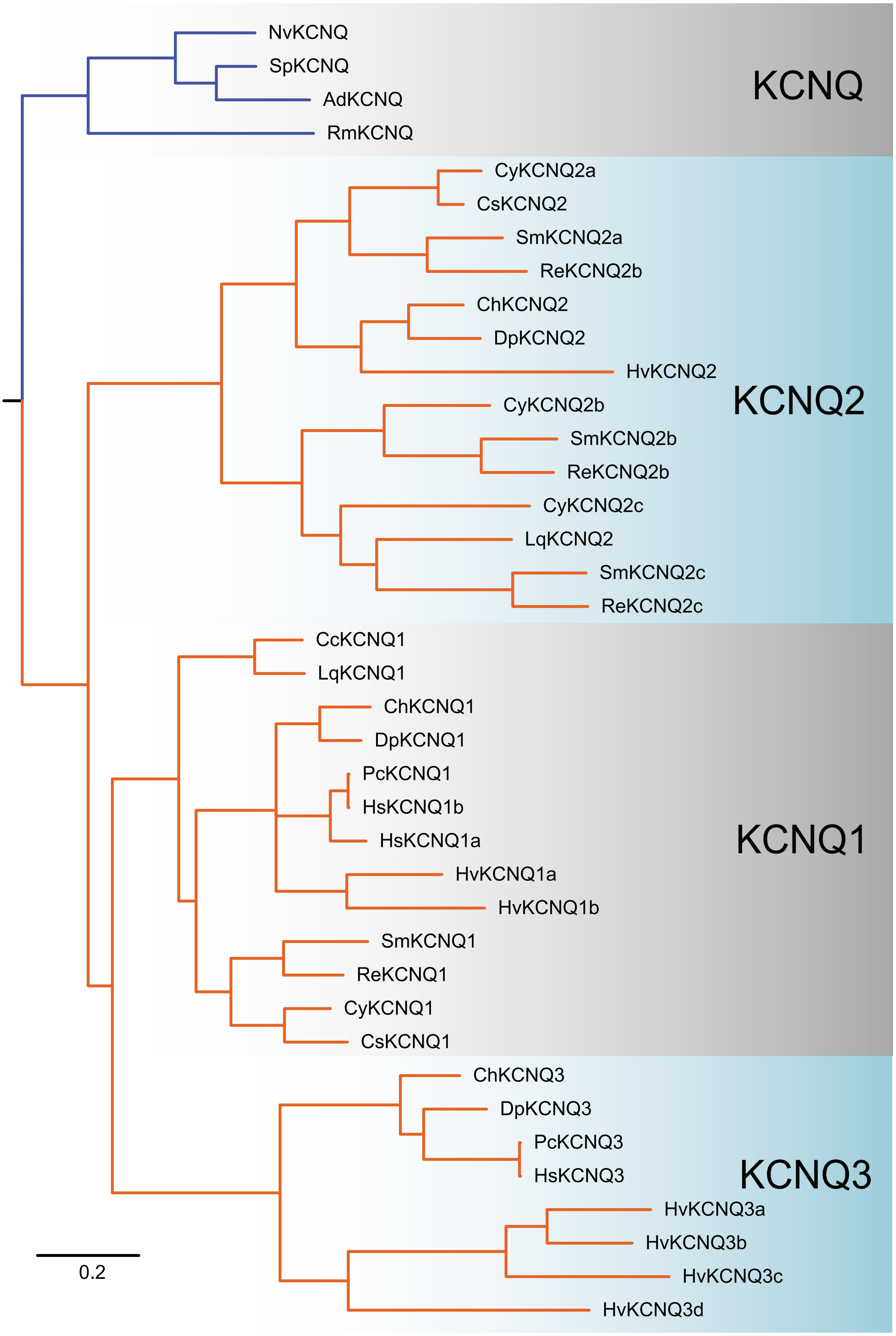
KCNQ
NvKCNQ
SpKCNQ
AdKCNQ
RmKCNQ
KCNQ2
CyKCNQ2a
CsKCNQ2
SmKCNQ2a
ReKCNQ2b
ChKCNQ2
DpKCNQ2
HvKCNQ2
CyKCNQ2b
SmKCNQ2b
ReKCNQ2b
CyKCNQ2c
LqKCNQ2
SmKCNQ2c
ReKCNQ2c
KCNQ1
CcKCNQ1
LqKCNQ1
ChKCNQ1
DpKCNQ1
PcKCNQ1
HsKCNQ1b
HsKCNQ1a
HvKCNQ1a
HvKCNQ1b
SmKCNQ1
ReKCNQ1
CyKCNQ1
CsKCNQ1
KCNQ3
ChKCNQ3
DpKCNQ3
PcKCNQ3
HsKCNQ3
HvKCNQ3a
HvKCNQ3b
HvKCNQ3c
HvKCNQ3d
0.2

Fig. S2

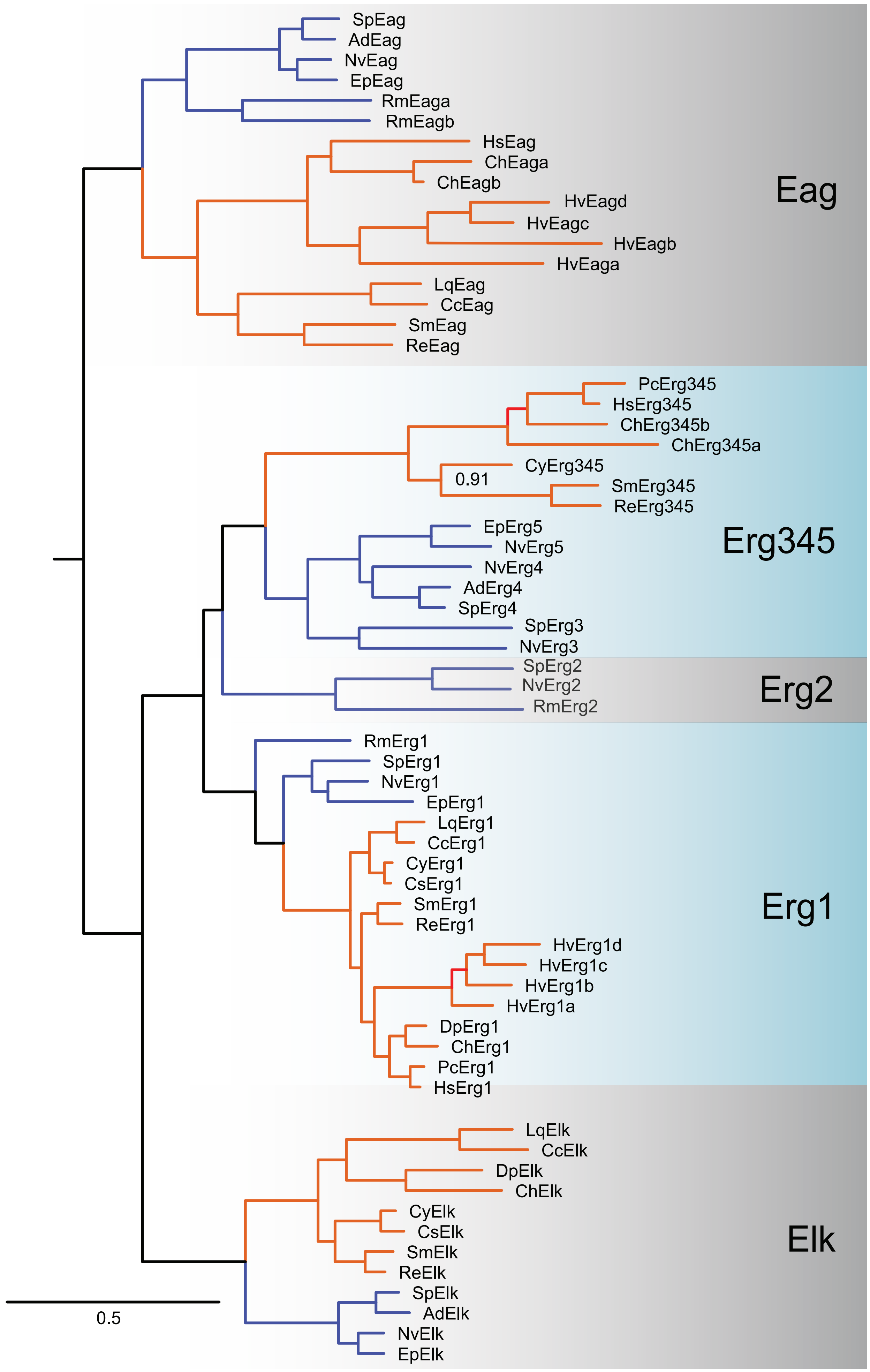
SpEag
AdEag
NvEag
EpEag
RmEaga
RmEagb
HsEag
ChEaga
ChEagb
HvEagd
HvEagc
HvEagb
HvEaga
LqEag
CcEag
SmEag
ReEag
Eag
PcErg345
HsErg345
ChErg345b
ChErg345a
CyErg345
0.91
SmErg345
ReErg345
EpErg5
NvErg5
NvErg4
AdErg4
SpErg4
SpErg3
NvErg3
Erg345
SpErg2
NvErg2
RmErg2
Erg2
RmErg1
SpErg1
NvErg1
EpErg1
LqErg1
CcErg1
CyErg1
CsErg1
SmErg1
ReErg1
HvErg1d
HvErg1c
HvErg1b
HvErg1a
DpErg1
ChErg1
PcErg1
HsErg1
Erg1
LqElk
CcElk
DpElk
ChElk
CyElk
CsElk
SmElk
ReElk
SpElk
AdElk
NvElk
EpElk
Elk
0.5

Fig. S3

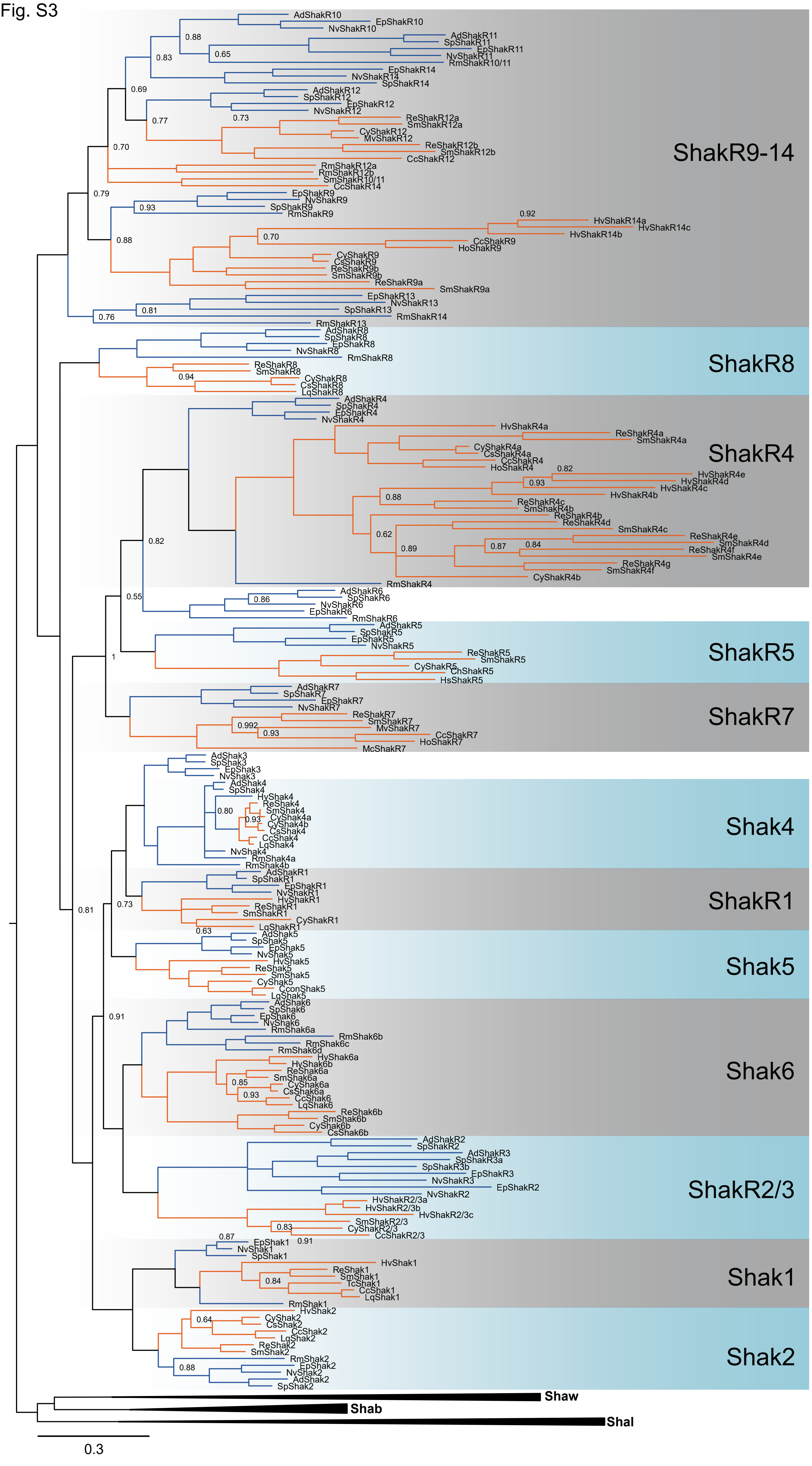

AdShakR10
EpShakR10
NvShakR10
0.88
AdShakR11
SpShakR11
0.83
0.65
EpShakR11
NvShakR11
RmShakR10/11
EpShakR14
NvShakR14
SpShakR14
0.69
AdShakR12
SpShakR12
EpShakR12
NvShakR12
0.77
0.73
ReShakR12a
SmShakR12a
CyShakR12
MvShakR12
0.70
ReShakR12b
SmShakR12b
CcShakR12
RmShakR12a
RmShakR12b
SmShakR10/11
CcShakR14
0.79
EpShakR9
NvShakR9
0.93
SpShakR9
RmShakR9
0.92
HvShakR14a
HvShakR14c
HvShakR14b
0.88
0.70
CcShakR9
HoShakR9
CyShakR9
CsShakR9
ReShakR9b
SmShakR9b
ReShakR9a
SmShakR9a
EpShakR13
NvShakR13
0.81
SpShakR13
0.76
RmShakR14
RmShakR13
ShakR9-14
AdShakR8
SpShakR8
EpShakR8
NvShakR8
RmShakR8
ReShakR8
SmShakR8
0.94
CyShakR8
CsShakR8
LqShakR8
ShakR8
AdShakR4
SpShakR4
EpShakR4
NvShakR4
HvShakR4a
ReShakR4a
SmShakR4a
CyShakR4a
CsShakR4a
CcShakR4
HoShakR4
ShakR4
0.82
HvShakR4e
HvShakR4d
0.93
HvShakR4c
HvShakR4b
0.88
ReShakR4c
SmShakR4b
ReShakR4b
ReShakR4d
0.82
SmShakR4c
0.62
ReShakR4e
SmShakR4d
0.87
0.84
ReShakR4f
0.89
SmShakR4e
ReShakR4g
SmShakR4f
CyShakR4b
RmShakR4
0.55
AdShakR6
SpShakR6
0.86
NvShakR6
EpShakR6
RmShakR6
AdShakR5
SpShakR5
EpShakR5
NvShakR5
ReShakR5
SmShakR5
1
CyShakR5
ChShakR5
HsShakR5
ShakR5
AdShakR7
SpShakR7
EpShakR7
NvShakR7
ReShakR7
SmShakR7
0.992
MvShakR7
0.93
CcShakR7
HoShakR7
McShakR7
ShakR7
AdShak3
SpShak3
EpShak3
NvShak3
AdShak4
SpShak4
HvShak4
ReShak4
SmShak4
0.80
CyShak4a
0.93
CyShak4b
CsShak4
CcShak4
LqShak4
NvShak4
RmShak4a
RmShak4b
Shak4
AdShakR1
SpShakR1
EpShakR1
NvShakR1
HvShakR1
ReShakR1
SmShakR1
CyShakR1
0.81
0.73
LqShakR1
ShakR1
0.63
AdShak5
SpShak5
EpShak5
NvShak5
HvShak5
ReShak5
SmShak5
CyShak5
CconShak5
LqShak5
Shak5
AdShak6
SpShak6
EpShak6
NvShak6
0.91
RmShak6a
RmShak6b
RmShak6c
RmShak6d
HvShak6a
HvShak6b
ReShak6a
SmShak6a
0.85
CyShak6a
CsShak6a
0.93
CcShak6
LqShak6
ReShak6b
SmShak6b
CyShak6b
CsShak6b
Shak6
AdShakR2
SpShakR2
AdShakR3
SpShakR3a
SpShakR3b
EpShakR3
NvShakR3
EpShakR2
NvShakR2
HvShakR2/3a
HvShakR2/3b
HvShakR2/3c
SmShakR2/3
CyShakR2/3
0.83
CcShakR2/3
ShakR2/3
0.87
EpShak1
0.91
NvShak1
SpShak1
HvShak1
ReShak1
SmShak1
0.84
TcShak1
CcShak1
LqShak1
RmShak1
Shak1
HvShak2
0.64
CyShak2
CsShak2
CcShak2
LqShak2
ReShak2
SmShak2
RmShak2
EpShak2
0.88
NvShak2
AdShak2
SpShak2
Shak2
Shaw
Shab
Shal
0.3

Fig. S4

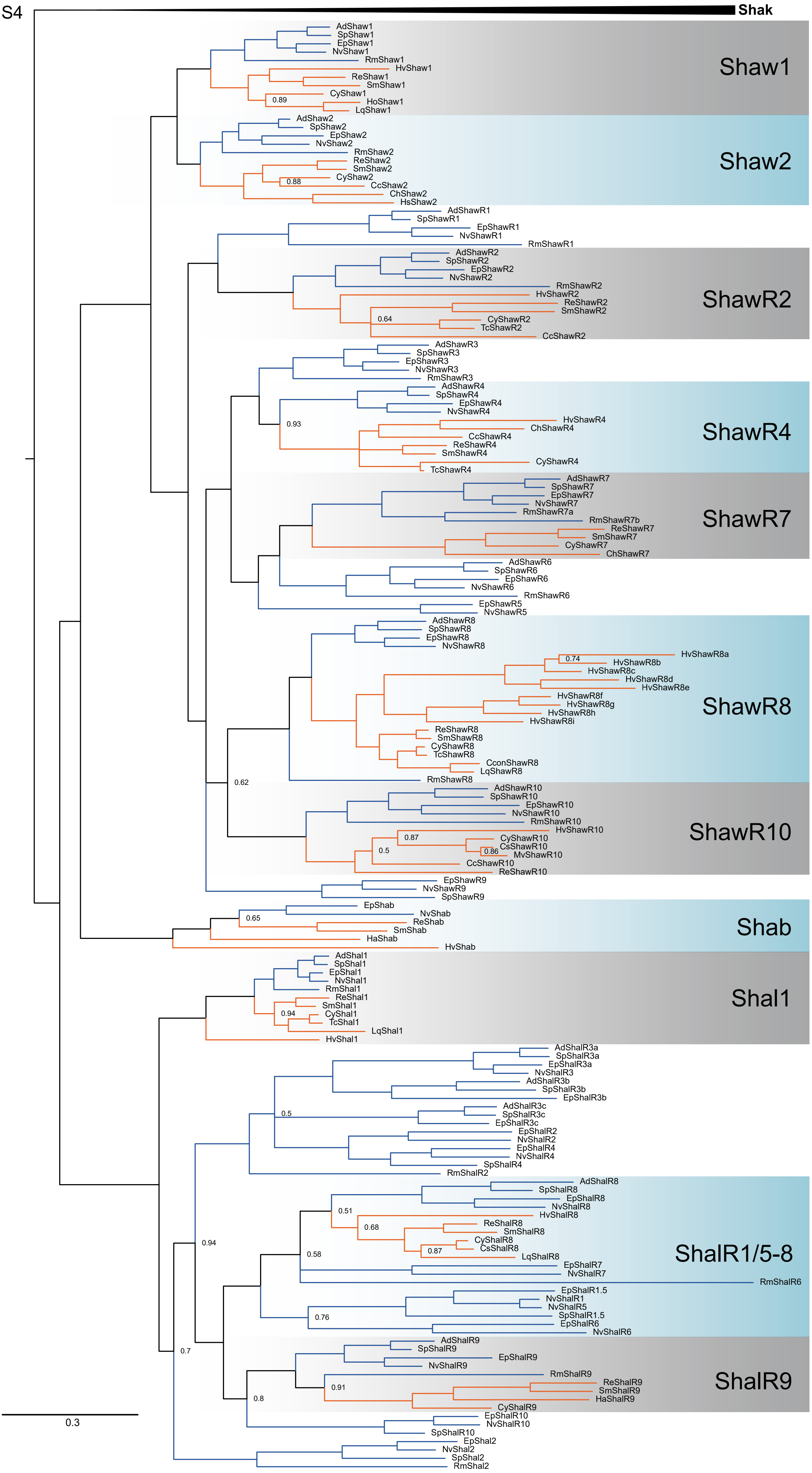
Shak
AdShaw1
SpShaw1
EpShaw1
NvShaw1
RmShaw1
HvShaw1
ReShaw1
SmShaw1
CyShaw1
0.89
HoShaw1
LqShaw1
Shaw1
AdShaw2
SpShaw2
EpShaw2
NvShaw2
RmShaw2
ReShaw2
SmShaw2
CyShaw2
0.88
CcShaw2
ChShaw2
HsShaw2
Shaw2
AdShawR1
SpShawR1
EpShawR1
NvShawR1
RmShawR1
AdShawR2
SpShawR2
EpShawR2
NvShawR2
RmShawR2
HvShawR2
ReShawR2
SmShawR2
0.64
CyShawR2
TcShawR2
CcShawR2
ShawR2
AdShawR3
SpShawR3
EpShawR3
NvShawR3
RmShawR3
AdShawR4
SpShawR4
EpShawR4
NvShawR4
0.93
HvShawR4
ChShawR4
CcShawR4
ReShawR4
SmShawR4
CyShawR4
TcShawR4
ShawR4
AdShawR7
SpShawR7
EpShawR7
NvShawR7
RmShawR7a
RmShawR7b
ReShawR7
SmShawR7
CyShawR7
ChShawR7
ShawR7
AdShawR6
SpShawR6
EpShawR6
NvShawR6
RmShawR6
EpShawR5
NvShawR5
AdShawR8
SpShawR8
EpShawR8
NvShawR8
HvShawR8a
0.74
HvShawR8b
HvShawR8c
HvShawR8d
HvShawR8e
HvShawR8f
HvShawR8g
HvShawR8h
HvShawR8i
ReShawR8
SmShawR8
CyShawR8
TcShawR8
CconShawR8
LqShawR8
RmShawR8
ShawR8
0.62
AdShawR10
SpShawR10
EpShawR10
NvShawR10
RmShawR10
HvShawR10
0.87
CyShawR10
CsShawR10
0.5
0.86
MvShawR10
CcShawR10
ReShawR10
ShawR10
EpShawR9
NvShawR9
SpShawR9
EpShab
0.65
NvShab
ReShab
SmShab
HaShab
HvShab
Shab
AdShal1
SpShal1
EpShal1
NvShal1
RmShal1
ReShal1
SmShal1
0.94
CyShal1
TcShal1
LqShal1
HvShal1
Shal1
AdShalR3a
SpShalR3a
EpShalR3a
NvShalR3
AdShalR3b
SpShalR3b
EpShalR3b
AdShalR3c
0.5
SpShalR3c
EpShalR3c
EpShalR2
NvShalR2
EpShalR4
NvShalR4
SpShalR4
RmShalR2
AdShalR8
SpShalR8
EpShalR8
NvShalR8
0.51
HvShalR8
ReShalR8
0.68
SmShalR8
0.94
CyShalR8
0.87
CsShalR8
0.58
LqShalR8
EpShalR7
NvShalR7
ShalR1/5-8
RmShalR6
EpShalR1.5
NvShalR1
NvShalR5
0.76
SpShalR1.5
EpShalR6
NvShalR6
AdShalR9
SpShalR9
EpShalR9
0.7
NvShalR9
RmShalR9
ReShalR9
0.91
SmShalR9
0.8
HaShalR9
CyShalR9
ShalR9
EpShalR10
NvShalR10
SpShalR10
EpShal2
NvShal2
SpShal2
RmShal2
0.3
